# The anti-predator role of within-nest emergence synchrony in sea turtle hatchlings

**DOI:** 10.1098/rspb.2016.0697

**Published:** 2016-07-13

**Authors:** Robson G. Santos, Hudson Tercio Pinheiro, Agnaldo Silva Martins, Pablo Riul, Soraya Christina Bruno, Fredric J. Janzen, Christos C. Ioannou

**Affiliations:** 1 Instituto de Ciências Biológicas e da Saúde, Universidade Federal de Alagoas, Maceió, Alagoas, Brazil; 2 Department of Ecology and Evolutionary Biology, University of California Santa Cruz, Santa Cruz, CA, USA; 3 California Academy of Sciences, San Francisco, CA, USA; 4 Departamento de Oceanografia e Ecologia, Universidade Federal do Espírito Santo, Vitória, Espirito Santo, Brazil; 5 Departamento de Engenharia e Meio Ambiente, CCAE, Universidade Federal da Paraíba, Rio Tinto, Paraíba, Brazil; 6 Fundação Pró-Tamar, Escritório Regional de Vitória, Vitória, Espirito Santo, Brazil; 7 Department of Ecology, Evolution, and Organismal Biology, Iowa State University, Ames, IA, USA; 8 School of Biological Sciences, University of Bristol, Bristol BS8 1TQ, UK

**Keywords:** sea turtles, anti-predator behaviour, predation risk, synchronous hatching, attack abatement, dilution effect

## Abstract

Group formation is a common behaviour among prey species. In egg-laying animals, despite the various factors that promote intra-clutch variation leading to asynchronous hatching and emergence from nests, synchronous hatching and emergence occurs in many taxa. This synchrony may be adaptive by reducing predation risk, but few data are available in any natural system, even for iconic examples of the anti-predator function of group formation. Here, we show for the first time that increased group size (number of hatchlings emerging together from a nest) reduces green turtle (*Chelonia mydas*) hatchling predation. This effect was only observed earlier in the night when predation pressure was greatest, indicated by the greatest predator abundance and a small proportion of predators preoccupied with consuming captured prey. Further analysis revealed that the effect of time of day was due to the number of hatchlings already killed in an evening; this, along with the apparent lack of other anti-predatory mechanisms for grouping, suggests that synchronous emergence from a nest appears to swamp predators, resulting in an attack abatement effect. Using a system with relatively pristine conditions for turtle hatchlings and their predators provides a more realistic environmental context within which intra-nest synchronous emergence has evolved.

## 1. Introduction

Individuals aggregating in temporary or permanent groups is a common behaviour among many species. These aggregations may be driven by a variety of reasons, such as defence, foraging and movement efficiency, and considerable attention has been focused on examining the benefits and costs of group formation [1]. Of the proposed explanations for grouping, reducing predation risk is perhaps the most general, and is widely believed to be one of the main drivers in the evolution of aggregation behaviour [2–4]. The relationship between prey group size and predation risk has been the target of many studies in a variety of species. Although costs arise from increased conspicuousness [5–7] (although see [8]), aggregation provides benefits because risk is diluted among group members [9], multiple targets visible simultaneously can confuse predators' targeting [10], and predators are more likely to be detected sooner through collective vigilance [11].

The synchrony of sea turtle hatchlings emerging from within a nest is typically believed to reduce predation [12–14] and is often used as a typical example of the anti-predator role of grouping [15] because predation on these otherwise helpless hatchlings is high as they crawl to the sea and swim away from the shore [16,17]. However, studies quantifying hatchling predation are scarce, especially during their crawl from their nests toward the sea [18,19]. Despite the suggestion that synchrony in sea turtle emergence is effective as an anti-predatory strategy, this hypothesis remains to be tested [20]. Peterson *et al*. [19], using freshwater turtles as a proxy for sea turtle hatchlings, found a decrease in the *per capita* predation risk with increased group size. Studying predation on a natural system (albeit one under anthropogenic disturbance), Tomillo *et al*. [18] found that the number of leatherback hatchlings (*Dermochelys coriacea*) killed by predators had a positive relationship with the number of hatchlings in an emergence. However, they did not present the relationship between *per capita* risk and group size, leaving it unclear whether dilution counterbalanced the suggested increased encounter rate with predators [20]. Thus, neither of these previous studies demonstrates that synchrony in emerging from a sea turtle nest has an anti-predator role, and it thus remains unknown whether the net effect of aggregation is to decrease *per capita* predation risk in natural systems [20].

Identifying the mechanism(s) that reduces risk in groups can be a challenging task, especially in observational studies of natural systems, due to limitations on monitoring behavioural interactions and control over possible confounding effects [21,22]. For example, while the confusion effect involves predators reducing their rate of attacks or success due to difficultly in targeting [23], and group vigilance relies on coordinated escape responses by prey after predator detection [11], both result in a decrease in *per capita* risk. As with Foster & Treherne's [9,24] classic water strider (*Halobates robustus*)–fish predator system, however, the potential mechanisms that could reduce risk for synchronously emerging sea turtles are limited. The confusion effect is unlikely to be important as most hatchings and emergences are nocturnal, so that visual cues are limited. Inter-individual cues between hatchlings that could transfer information about the presence of a predator, a requirement for group vigilance, have not been observed and neither have any collective defence strategies. Thus, a likely mechanism is attack abatement [4], which relies on an encounter rate with predators that does not increase as quickly as (or faster than) group size [8], and a dilution effect, which limits the number of prey that are eaten in each encounter [9]. The ‘swamping’ of predators by synchronous emergence when hatching may occur due to the highly limited consumption rate of the hatchlings' main terrestrial predator in our study area, the yellow crab (*Johngarthia lagostoma*), as the size of these predators (adults' carapace lengths: 60–120 mm [25]) is relatively close to the typical size of a green turtle hatchling (carapace length: 50 mm [26]). Thus, handling times are expected to be relatively long when a crab captures a hatchling. It is also unlikely that these predators respond quickly enough to a nest emergence so that their encounter rate with the group is proportional to group size due to the wide distribution of nests over the beach and the limited range over which prey can be detected. Thus, the conditions necessary for attack abatement may be met when sea turtles emerge synchronously, and this would be the first demonstration of attack abatement in a vertebrate prey.

Damage to coastal habitats due to anthropogenic activities is so pervasive that opportunity to study and understand natural ecological and evolutionary interactions in coastal communities is rapidly waning [27,28]. Here, we investigated in a natural system how group size (i.e. the number of hatchlings emerging together from a nest) influences predation on green turtle (*Chelonia mydas*) hatchlings. Synchrony can also occur in hatching (before emergence) and between nests laid by different females; our study only concerns synchrony of emergence from a nest (‘within’ nest synchrony). We conducted our study on an oceanic island (Trindade Island, Brazil) that offers relatively pristine conditions for green turtle hatchlings and the yellow crab. The low level of anthropogenic disturbance in this beach environment provides a system that should be relatively representative of the conditions under which intra-nest synchronous emergence evolved.

## 2. Material and methods

### (a) Study area

Trindade is a volcanic island uplifted 3–3.5 million years ago [29,30], with a total area of 9.2 km^2^ and a narrow platform (0–50 m depth) [31]. It is located approximately 1200 km east of mainland Brazil (20°30′ S; 29°20′ W), with a Brazilian Navy settlement since 1957. Trindade is considered the only Brazilian nesting site that has not suffered hunting of female *C. mydas* in recent times. The island is the main nesting ground for green sea turtles in Brazil, hosting approximately 3600 nests y^−1^ on just 3 km of sand beaches, and is among the most important known rookeries in the Atlantic system for green turtles [32,33]. Thus, our study area is a sample of a large population, rather than being a marginal site that may not be representative of nesting grounds for this species. The green sea turtle is the only chelonian that nests on the island and the peak season is January–March [34]. Since 1982, TAMAR-ICMBio has regularly monitored *C. mydas* nests in Trindade. Our study was conducted on Tartarugas beach (300 m in length), the main nesting beach on the island.

### (b) Nests and hatchlings group size

We monitored 33 green sea turtle nests that were laid in February and March 2009. We placed a circular plastic-mesh corral (50 cm diameter, 50 cm height, 1 cm mesh size) around each nest 40 days after egg deposition to prevent emergent hatchlings from dispersing. This timing was calculated based on incubation durations of nests recorded in previous seasons (43–77 days; TAMAR-ICMBio database). We did not disturb the nests once they were encircled with mesh, allowing hatchlings to emerge without assistance.

We visually checked nests every half an hour throughout the study from 17.30 to 06.00 every night. The corrals remained open from 06.00 to 17.00 to avoid hatchling desiccation in case of diurnal emergence. We checked nests four times daily (10.00, 12.00, 14.00 and 16.00) to count tracks of emerged hatchlings, but these groups were not included in the analysis. We checked the integrity of the corrals constantly during the study period to ensure that no hatchling escaped.

We recorded the following variables to assess group sizes and timing for each emergence from a nest: the order of the emergence event within a nest, the number of hatchlings in each emergence event (group size) and the time of emergence events (hours). We identified an emergence event if at least one hatchling emerged. When we identified an emergence event, we waited 10 min from the emergence of the last hatchling to ensure that the emergence event was concluded.

### (c) Predation

The extant terrestrial fauna of Trindade Island is formed by an unknown number of insect and arachnid species, seabirds, the yellow crab (*J. lagostoma*), the introduced tropical house gecko (*Hemidactylus mabouia*) and mice (*Mus musculus*) [34]. Among all the extant terrestrial fauna, yellow crabs are the most abundant nocturnal terrestrial animal capable of predating green turtle hatchlings. Therefore, we evaluated predation on land focusing on the most abundant predator, the yellow crab [34,35]. The yellow crab's absence of a behavioural response to human presence in Trindade Island is long recognized [35]; this naivety is probably due to the virtual lack of predators when individuals reach the adult phase. This behaviour of yellow crabs in Trindade Island helps to minimize any effect of the observers on predator behaviour in our study. Most of the yellow crabs do not live on the beach; they live in burrows on upper vegetated areas and crawl to the beach at night to search for food. Typically, they will feed each night, given the opportunity; thus, we believe all crabs observed in the surveys were either actively searching for, or consuming, food. During all the field activities we did not find these crabs engaging in any other behaviours during the night (e.g. reproduction). To quantify crab abundance, we used three parallel 50 m transects, 100 m apart, starting at the high tide line and running inland. We conducted surveys during three time periods (17.30–21.00, 21.00–01.00 and 01.00–05.00) for seven nights during the emergence period of most of the nests (late April to early May). We counted all crabs detected within 3 m of a transect and the number of crabs that had captured a sea turtle hatchling. We considered a crab to have captured prey when we found it holding a hatchling. Owing to the large size of the prey relative to the predators, handling times of the prey are long and it is difficult for the crabs to move prey from where they are caught, so they are consumed close to the point of capture.

After swiftly counting the hatchlings from an emergence event at a nest site, we turned off our flashlights and released the turtles, allowing them to continue freely crawling toward the sea. We waited a set time until the neonates reached the sea before we turned on the flashlights and searched for depredated hatchlings. We calculated the waiting time based on the distance from the nest to the tide line and a hatchling crawling speed of 5 m min^−1^ (*sensu* Dial [36]). The search for depredated hatchlings was conducted by two observers within 5 m of a transect from the nest to the tide line. To ensure that we counted hatchlings only from a focal nest, we searched the transect area for non-target *C. mydas* prior to releasing the hatchlings.

### (d) Statistical analyses

**Figure 1.**
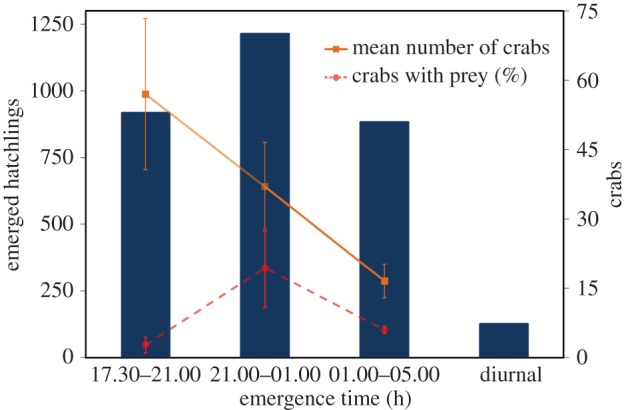
Temporal distribution of emerged green turtle hatchlings (columns, data from the nest emergences); mean (±s.e.) density of yellow crabs at night (solid line) and mean (±s.e.) relative number of crabs that have captured a green turtle hatchling (dashed line, data from crab surveys). (Online version in colour.)

The total number of crabs in each survey was analysed as a function of time period (the middle time was used for each period, i.e. 19.15, 23.00 and 03.00) using a generalized linear model (GLM) with a negative binomial error distribution. The proportion of crabs that captured a turtle hatchling was also analysed as a function of time period with the polynomial effect of time included after visually inspecting the data (figure 1). A GLM with a quasi-binomial error distribution was used due to overdispersion.

The number of hatchlings in an emergence event (i.e. group size) was analysed as a function of the time of day, date, the distance from the nest to the high tide line and the order of emergence within that nest. The analyses were thus carried out at the level of the emergence (i.e. group, *n* = 51), rather than at the level of the nest (*n* = 33). Two-way interactions between emergence order and each of the other variables were included. A generalized linear mixed model (GLMM) with a negative binomial error distribution was used. To test for significant effects, each term was removed in turn from the model and compared with the model including this term. We removed the least significant two-way interactions in each model (on the condition that *p* > 0.1) before repeating the process with the remaining terms. All main effects remained in the final model as control variables.

Predation risk was quantified as the number of hatchlings killed as a proportion of the number of hatchlings in each emergence event from a nest. We used a GLMM with a binomial error distribution (glmmPQL was used as the data were overdispersed) to test the effects of group size, time of day, date and the distance from the nest to the high tide line, with two-way interactions included between group size and each of the other variables (non-significant interactions were removed as above). To further explore predation risk, we calculated the number of depredated hatchlings found in an evening before the emergence of each group and repeated the analysis of predation risk per group with this information as an additional explanatory variable.

Nest was included as a random variable in the GLMMs, as multiple emergence events were recorded from some nests. In the analyses, time of day was converted from the 24 h clock to time elapsed since 00.00 the previous night (e.g. 03.00 was coded as 27 h). The date was converted in a similar manner from the first date of data collection. All analyses were performed in R v. 2.15.1 [37].

## 3. Results

### (a) Prey: green sea turtles hatchling emergence

**Figure 2.**
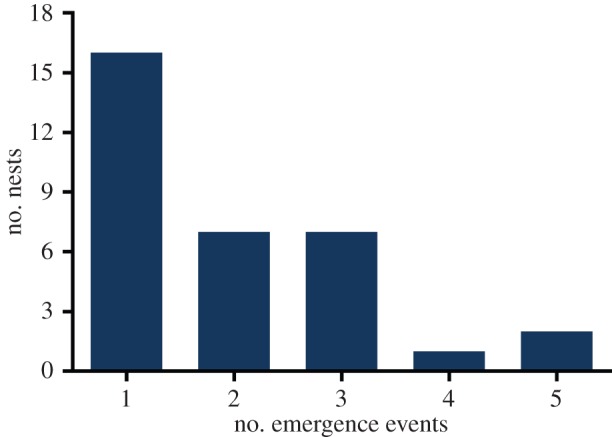
Number of emergence events per nest for the 33 green turtle nests from Trindade Island, Brazil. (Online version in colour.)

A total of 3177 green sea turtle hatchlings emerged from the 33 monitored nests during the study. The vast majority of hatchlings emerged at night (figure 1). Diurnal emergence did occur for two *C. mydas* nests and accounted for only 3.7% of total emerged hatchlings. We observed and recorded data from 2494 hatchlings in 51 groups. It was not possible to evaluate eight groups (683 hatchlings) due to logistical problems such as storms. From the first emergence to the last, 21 days transpired, with 2.2 groups per night on average. Most nests produced all hatchlings within a single group (figure 2), and in cases where multiple groups emerged from the same nest, the number of hatchlings decreased significantly in subsequent emergences (negative binomial GLMM: deviance_4,5_ = 52.80, *p* = 3.69 × 10^−13^). The number of hatchlings per emergence (group size) also tended to increase as the season progressed (deviance_4,5_ = 4.92, *p* = 0.026), with distance to the sea and the time of day having no effect (*p* > 0.5 in both cases). From all groups that emerged on the same night, only in seven occasions were the groups less than 2 h apart. Additionally, on these occasions, the smallest distance between nests was 27.8 m (mean = 86.6 m), which makes interactions between groups unlikely. Group size varied from 1 to 175 individuals, with an average of 48.9 (s.e. ± 7.6) hatchlings per group.

### (b) Predator: yellow crab

The mean density of yellow crabs was 3.70 ± 2.04 crabs 100 m^−2^ (range = 1.52–6.67 crabs). Based on beach length (300 m) and distance from the farthest nest to the high tide line (50 m), the mean number of crabs was more than 500 per night. Crab numbers were highest early in the evening and declined during the night (figure 1; negative binomial GLM: LRT_1,15_ = 7.56, *p* = 0.0060), and the proportion of crabs that were found to have captured a hatchling peaked in the middle time period of 21.00–01.00 (figure 1; quasi-binomial GLM, polynomial effect of time: *F*_2,14_ = 5.95, *p* = 0.013). This suggests a delay for the predators in becoming active and actually finding prey to consume. Therefore, the number of crabs actively searching for food, and hence representing a risk of predation to emerging hatchlings, was much greater at the start of the night (17.30–21.00) compared with any other time.

### (c) Predation

**Figure 3.**
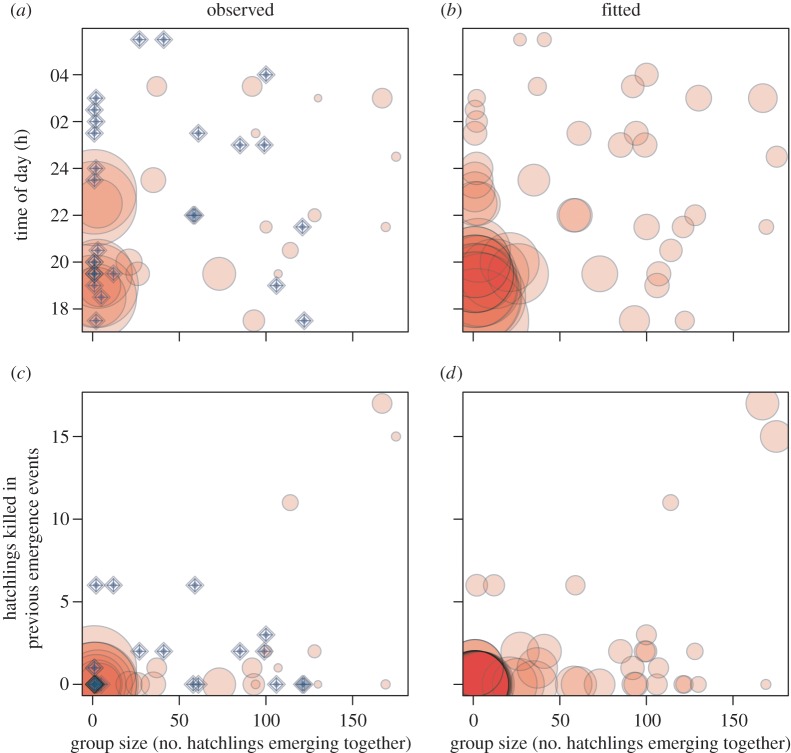
Determinants of predation risk in green turtle hatchlings. *Per capita* predation risk is represented by bubble area; groups without any mortality (i.e. zero risk) are represented by diamonds. Risk is plotted against group size and (*a*,*b*) time of day or (*c*,*d*) number of prey already killed that evening. (*a*) and (*c*) show the observed risk per group, while (*b*) and (*d*) show the fitted (i.e. predicted) risk from GLMMs with the two axes as interacting covariates and nest as a random factor. (Online version in colour.)

From all 2494 hatchlings, 2.65% were depredated by crabs prior to reaching the sea. In the analysis of predation risk, only the interaction between group size and time of day was significant (GLMM: *F*_1,16_= 7.59, *p* = 0.014), with date and distance from the sea having non-significant interactions with group size and main effects (*p* > 0.2 in all cases). The significant interaction was due to predation risk being greater for smaller groups, but only earlier in the evening (figure 3*a*,*b*).

To explore why the time of day affected predation risk in small groups, we calculated the number of depredated hatchlings found that evening before the emergence of each group. Although positively related to the time of day as expected, the two variables were not collinear (Spearman's rank: *r*_s_ = 0.43, *p* = 0.0014). When this variable and its interaction with group size was included in the model explaining predation risk, the previously significant interaction between group size and time became non-significant (GLMM: *F*_1,12_ = 0.47, *p* = 0.51), while the interaction between group size and number of hatchlings already depredated was significant (*F*_1,15_ = 6.20, *p* = 0.025; all other effects *p* > 0.1). Thus, the effect of time of day on the safety provided by groups could, at least partially, be explained by the number of hatchlings already killed and consumed that evening (figure 3*c*,*d*).

## 4. Discussion

Our study reveals a pattern of highly synchronous nocturnal emergence within nests, with hatchlings in the majority of nests departing in a single emergence event. The nocturnal emergence will prevent death by overheating and desiccation, and decrease predation by visual and diurnal predators, such as seabirds [38,39]. Emergence synchrony is predicted to be favoured by natural selection [40] because mass departure with large groups of hatchlings should saturate the foraging ability of predators, thereby reducing the predation threat to individuals [12]. Predator satiation is used to explain breeding aggregations that are unpredictable to predators in time and/or space, such as the mast seeding of some plants [41], and large aggregations of invertebrates [42,43] and vertebrates [44,45]. Although the large groups formed by sea turtle hatchlings during their emergence from nests have long been predicted to be an anti-predator strategy [15], the relationship between their group size and predation risk remained unknown [20]. Our results provide evidence for this hypothesis: risk was reduced in larger groups, at least early in the evening when the main predator (the yellow crab) was most abundant, and also unlikely to already be handling and consuming prey.

It has been argued that the risk of detection (i.e. predator avoidance) and the risk of being attacked (i.e. the dilution effect) cannot be considered separately; only the combination of the two will determine if group living reduces predation risk (the attack abatement effect) [4]. However, it is often difficult to isolate predator avoidance and dilution effects from other anti-predatory grouping mechanisms. Of the few explicit empirical studies of attack abatement, none have used a vertebrate prey species [6,46,47]. In our system, the highly stereotyped behaviour of hatchlings crawling towards the sea shows no indication of information transfer among individuals, which excludes coordinated evasive behaviour such as the ‘many eyes' effect. The very limited visibility at night and the small visual range of the main predator relative to the spatial extent of the prey group also makes a confusion effect highly unlikely. The decrease in risk with increased group size may instead be best explained by attack abatement, which relies on an encounter rate with predators that does not increase as fast as (or faster than) group size [8], and a dilution effect, which limits the number of prey that are killed in each encounter [9]. The unpredictable and ephemeral availability of hatchlings and the limited ability of the crabs to detect hatchlings from far away should result in a sub-linear (or no) increase in predation relative to group size, a pattern that is widespread [8,32,43,48], even in conspicuous prey [5]. Additionally, the size of the predator relative to the prey limits the number of prey consumed per predator per night due to long handling times [49]. These effects are supported by our results, which show a delay between the highest abundance of hatchlings and the peak in the proportion of crabs found with prey, suggesting crabs took some time to locate and kill prey, and the importance of the number of prey already killed in a night on predation risk, suggesting substantial handling times once prey had been found (leading to predator swamping). To demonstrate an attack abatement mechanism more directly, behavioural interactions between hatchlings and crabs could be monitored, for example using infrared lighting or GPS units on crabs to investigate how crabs respond to an emergence from a nest and how their foraging behaviour changes once a hatchling is captured.

Although our study focused on synchrony of emergence within nests, our results also raise interesting questions regarding the role of female nesting synchrony (i.e. synchrony between nests), and more generally about the interactions between multiple groups regarding when to time exposure to predators. Female nesting synchrony should be favoured to maximize the number of prey available, and thus swamp predators [14,50], although predation is only one of the potential selective agents that may affect the evolution of reproductive strategies [3]. However, few attempts have been made to test the predator-swapping hypothesis [50]. The effects of predator satiation may be stronger for hatchlings that emerge from nests deposited during the peak of the nest season, where 75% of the nests were recorded during 56 days (TAMAR-ICMBio dataset; also see [33]). However, emerging later within an evening was associated with a decrease in risk, particularly for hatchlings emerging in smaller group sizes, due to fewer predators and an increase in the proportion of those already preoccupied with prey. This result suggests that delaying emergence, rather than synchrony, would be advantageous at the scale within the evening. Other factors, such as loss of energy due to catabolism of residual yolk [51,52] and risk of desiccation associated with late emergences [53], would need to be considered, as well as local abundance of both prey and predators. A modelling approach would thus be useful to guide further investigations of these systems (e.g. [45]).

Synchronous emergence is commonly reported to be an anti-predatory behaviour for many species [3]. Synchronous hatching in turtles is common, and likely to be an ancestral trait [15,40,54], despite the different rates of development within single nests [13,55]. Our study reveals a pattern of high intra-nest synchronicity in emergence and its benefit as an anti-predator strategy for sea turtles. At a mechanistic level, synchrony may arise from social facilitation during ascent through the sand column, as hypothesized by Carr & Hirth [56] and Spencer *et al*. [13]. It is currently unknown whether individuals hatching in response to hatching nest-mates evolved to reduce risk via increased synchronous emergence, or whether it evolved for reasons other than anti-predator defence (i.e. an exaptation [57]). The timing of emergence may be influenced by other factors, such as physiological (e.g. oxygen levels [58]) and thermoregulatory constraints (e.g. thermal cues that signals hatchlings to emerge from the sand [38,39,59]). Intra-nest emergence synchrony is not universal in all sea turtle nesting areas [60]. More studies under different predation scenarios are needed to clarify this question. However, care must be taken in conducting such studies, because humans have altered most marine coastal ecosystems before modern ecological investigations began, and thus the present may not always be the key to the past [28].

## Data accessibility

Data presented in this paper are available as an electronic supplementary material accessible through Dryad (http://datadryad.org/review?doi=doi:10.5061/dryad.h4m68).

## References

[1] Krause J, Ruxton GD 2002 Living in groups. Oxford, UK: Oxford University Press.

[2] Hamilton WD 1971 Geometry for the selfish herd. J. Theor. Biol. 31, 295–311. (10.1016/0022-5193(71)90189-5) 5104951

[3] Ims RA 1990 On the adaptive value of reproductive synchrony as a predator-swamping strategy. Am. Nat. 136, 485–498. (10.1086/285109)

[4] Turner GF, Pitcher TJ 1986 Attack abatement: a model for group protection by combined avoidance and dilution. Am. Nat. 128, 228–240. (10.1086/284556)

[5] Riipi M, Alatalo RV, Lindstro L, Mappes J 2001 Multiple benefits of gregariousness cover detectability costs in aposematic aggregations. Nature 413, 512–514. (10.1038/35097061) 11586357

[6] Wrona FJ, Dixon RWJ 1991 Group size and predation risk: a field analysis of encounter and dilution effects. Am. Nat. 137, 186–201. (10.1086/674378)

[7] Ioannou CC, Ruxton GD, Krause J 2008 Search rate, attack probability, and the relationship between prey density and prey encounter rate. Behav. Ecol. 19, 842–846. (10.1093/beheco/arn038)

[8] Ioannou CC, Bartumeus F, Krause J, Ruxton GD 2011 Unified effects of aggregation reveal larger prey groups take longer to find. Proc. Biol. Sci. *B* 278, 2985–2990. (10.1098/rspb.2011.0003) 21325333 PMC3151713

[9] Foster WA, Treherne JE 1981 Evidence for the dilution effect in the selfish herd from fish predation on a marine insect. Nature 293, 466–467. (10.1038/293466a0)

[10] Ioannou CC, Morrell LJ, Ruxton GD, Krause J 2009 The effect of prey density on predators: conspicuousness and attack success are sensitive to spatial scale. Am. Nat. 173, 499–506. (10.1086/597219) 19231967

[11] Godin J-GJ, Classon LJ, Abrahams MV 1988 Group vigilance and shoal size in a small characin fish. Behaviour 104, 29–40. (10.2307/4534656)

[12] Dehn MM 1990 Vigilance for predators: detection and dilution effects. Behav. Ecol. Sociobiol. 26, 337–342. (10.1007/BF00171099)

[13] Spencer RJ, Thompson MB, Banks PB 2001 Hatch or wait? A dilemma in reptilian incubation. Oikos 93, 401–406. (10.1034/j.1600-0706.2001.930305.x)

[14] Tucker JK, Paukstis GL, Janzen FJ 2008 Does predator swamping promote synchronous emergence of turtle hatchling among nests? Behav. Ecol. 19, 35–40. (10.1093/beheco/arm097)

[15] Spencer R-J, Janzen FJ 2011 Hatching behavior in turtles. Integr. Comp. Biol. 51, 100–110. (10.1093/icb/icr045) 21659391

[16] Stancyk SE 1982 Non-human predators of sea turtles and their control. In Biology and conservation of sea turtles (ed. Bjorndal KA), pp. 139–152. Washington, DC: Smithsonian Institution Press.

[17] Frazer NB 1986 Survival from eggs to adulthood in a declining population of loggerhead turtles, *Caretta caretta*. Herpetologica 42, 47–55.

[18] Tomillo PS, Paladino FV, Suss JS, Spotila JR 2010 Predation of leatherback turtle hatchlings during the crawl to the water. Chelonian Conserv. Biol. 9, 18–25. (10.2744/CCB-0789.1)

[19] Peterson C, Fegley S, Voss C, Marschhauser S, VanDusen B 2013 Conservation implications of density-dependent predation by ghost crabs on hatchling sea turtles running the gauntlet to the sea. Mar. Biol. 160, 629–640. (10.1007/s00227-012-2118-z)

[20] Heithaus MR 2013 Predators, prey, and the ecological roles of sea turtles. In The biology of sea turtles, *vol. III* (eds Wyneken J, Lohmann KJ, Musick JA), pp. 249–284. Boca Raton, FL: CRC Press.

[21] Beauchamp G, Ruxton G 2008 Disentangling risk dilution and collective detection in the antipredator vigilance of semipalmated sandpipers in flocks. Anim. Behav. 75, 1837–1842. (10.1016/j.anbehav.2007.12.016)

[22] Cresswell W 1994 Flocking is an effective anti-predation strategy in redshanks, *Tringa totanus*. Anim. Behav. 47, 433–442. (10.1006/anbe.1994.1057)

[23] Ioannou CC, Tosh CR, Neville L, Krause J 2008 The confusion effect—from neural networks to reduced predation risk. Behav. Ecol. 19, 126–130. (10.1093/beheco/arm109)

[24] Treherne JE, Foster WA 1982 Group size and anti-predator strategies in a marine insect. Anim. Behav. 30, 536–542. (10.1016/S0003-3472(82)80066-3)

[25] Hartnoll RG, Mackintosh T, Pelembe TJ 2006 *Johngarthia lagostoma* (H. Milne Edwards, 1837) on Ascension Island: a very isolated land crab population. Crustaceana 79, 197–215. (10.1163/156854006776952900)

[26] National Marine Fisheries Service & U.S. Fish and Wildlife Service. 1991 Recovery plan for U.S. population of Atlantic green turtle. Washington, DC: National Marine Fisheries Service.

[27] Dayton PK 1998 Reversal of the burden of proof in fisheries management. Science 279, 821–822. (10.1126/science.279.5352.821)

[28] Jackson JBC 2001 What was natural in the coastal oceans? Proc. Natl Acad. Sci. USA 98, 5411–5418. (10.1073/pnas.091092898) 11344287 PMC33227

[29] Almeida FFM 1961 Geologia e petrologia da Ilha da Trindade. Monografia 18, 1–197.

[30] Greenwood JC 1998 Barian-titanian micas from Ilha da Trindade, South Atlantic. Mineral. Mag. 62, 687–695. (10.1180/002646198547918)

[31] Gasparini JL, Floeter SR 2001 The shore fishes of Trindade Island, western South Atlantic. J. Nat. Hist. 35, 1639–1656. (10.1080/002229301317092379)

[32] Seminoff JA 2004 *Chelonia mydas*. IUCN Red List of Threatened Species. Version 2014.3. See http://www.iucnredlist.org/details/4615/0.

[33] Almeida AP, Moreira LMP, Bruno SC, Thomq JCA, Martins AS, Bolten AB, Bjorndal KA 2011 Green turtle nesting on Trindade Island, Brazil: abundance, trends, and biometrics. Endanger. Species Res. 14, 193–201. (10.3354/esr00357)

[34] Alves RJV, da Silva NG, Aguirre-Muñoz A 2011 Return of endemic plant populations on Trindade Island, Brazil, with comments on the fauna. In Island invasives: eradication and management (eds Veitch CR, Clout MN, Towns DR), pp. 259–263. Gland, Switzerland: IUCN.

[35] Lobo B 1919 Conferência sobre a Ilha da Trindade. Arq. do Mus. Nac. Rio Janeiro 22, 107–170.

[36] Dial BE 1983 Energetics and performance during nest emergence and the hacthling frenzy in loggerhead sea turtles (*Caretta caretta*). Herpetologica 43, 307–315. (10.2307/3892496)

[37] R Development Core Team. 2011 R: a language and environment for statistical computing. Austria, Vienna: R Foundation for Statistical Computing.

[38] Mrosovsky N 1968 Nocturnal emergence of hatchling sea turtles: control by thermal inhibition of activity. Nature 220, 1338–1339. (10.1038/2201338a0) 5701356

[39] Drake DL, Spotila JR 2001 Thermal tolerances and the timing of sea turtle hatchling emergence. J. Therm. Biol. 27, 71–81. (10.1016/S0306-4565(01)00017-1)

[40] Glen F, Broderick AC, Godley BJ, Hays GC 2005 Patterns in the emergence of green (*Chelonia mydas*) and loggerhead (*Caretta caretta*) turtle hatchlings from their nests. Mar. Biol. 146, 1039–1049. (10.1007/s00227-004-1492-6)

[41] Kelly D 1994 The evolutionary ecology of mast seeding. Trends Ecol. Evol. 9, 465–470. (10.1016/0169-5347(94)90310-7) 21236924

[42] Sweeney BW, Vannote RL 1982 Population synchrony in mayflies: a predator satiation hypothesis. Evolution 36, 810–821. (10.2307/2407894) 28568232

[43] Williams KS, Smith KG, Stephen FM 1993 Emergence of 13-Yr periodical cicadas (Cicadidae: Magicicada): phenology, mortality, and predator satiation. Ecology 74, 1143–1152. (10.2307/1940484)

[44] Eckrich CE, Owens DW 1995 Solitary versus arribada nesting in the olive ridley sea turtles (*Lepidochelys olivacea*): a test of the predator-satiation hypothesis. Herpetologica 51, 349–354. (10.2307/3893041)

[45] Milner-Gulland EJ 2001 A dynamic game model for the decision to join an aggregation. Ecol. Model. 145, 85–99. (10.1016/S0304-3800(01)00381-7)

[46] Jensen K, Larsson P 2002 Predator evasion in *Daphnia*: the adaptive value of aggregation associated with attack abatement. Oecologia 132, 461–467. (10.1007/s00442-002-0979-4) 28547425

[47] Uetz GW, Hieber CS 1994 Group size and predation risk in colonial web-building spiders: analysis of attack abatement mechanisms. Behav. Ecol. 5, 326–333. (10.1093/beheco/5.3.326)

[48] Johannesen A, Dunn AM, Morrell LJ 2014 Prey aggregation is an effective olfactory predator avoidance strategy. PeerJ 2, e408 (10.7717/peerj.408) 24918032 PMC4045334

[49] Holling CS 1959 Some characteristics of simple types of predation and parasitism. Can. Entomol. 91, 385–398. (10.4039/Ent91385-7)

[50] Rolf Anker I 1990 The ecology and evolution of reproductive synchrony. Trends Ecol. Evol. 5, 135–140. (10.1016/0169-5347(90)90218-3) 21232341

[51] Hays GC, Speackman JR, Hayes JP, Speakman JR, Hayes JP 1992 The pattern of emergence by loggerhead turtle (*Caretta caretta*) hatchlings on Cephalonia, Greece. Herpetologica 48, 396–401.

[52] Godfrey MH, Mrosovsky N 1997 Estimating the time between hatching of sea turtles and their emergence from nest. Chelonian Conserv. Biol. 2, 581–585.

[53] Matsuzawa Y, Sato K, Sakamoto W, Bjorndal KA 2002 Seasonal fluctuations in sand temperature: effects on the incubation period and mortality of loggerhead sea turtle (*Caretta caretta*) pre-emergent hatchlings in Minabe, Japan. Mar. Biol. 140, 639–646. (10.1007/s00227-001-0724-2)

[54] Colbert PL, Spencer RJ, Janzen FJ 2010 Mechanism and cost of synchronous hatching. Funct. Ecol. 24, 112–121. (10.1111/j.1365-2435.2009.01602.x)

[55] Gyuris E 1993 Factors that control the emergence of green turtle hatchlings from the nest. Wildl. Res. 20, 345–353. (10.1071/WR9930345)

[56] Carr A, Hirth H 1961 Social facilitation in green turtle siblings. Anim. Behav. 9, 68–70. (10.1016/0003-3472(61)90051-3)

[57] Gould SJ, Vrba ES 1982 Exaptation; a missing term in the science of form. Paleobiology 8, 4–15. (10.1017/S0094837300004310)

[58] Ackerman RA 1980 Physiological and ecological aspects of gas exchange by sea turtle eggs. Am. Zool. 20, 575–583. (10.1093/icb/20.3.575)

[59] Glen F, Broderick AC, Godley BJ, Hays GC 2006 Thermal control of hatchling emergence patterns in marine turtles. J. Exp. Mar. Bio. Ecol. 334, 31–42. (10.1016/j.jembe.2006.01.005)

[60] Houghton JDR, Hays GC 2001 Asynchronous emergence by loggerhead turtle (*Caretta caretta*) hatchlings. Naturwissenschaften 88, 133–136. (10.1007/s001140100212) 11402844

